# Holistic word processing in dyslexia

**DOI:** 10.1371/journal.pone.0187326

**Published:** 2017-11-09

**Authors:** Aisling Conway, Nuala Brady, Karuna Misra

**Affiliations:** School of Psychology, University College Dublin, Belfield, Dublin, Ireland; University of Akron, UNITED STATES

## Abstract

People with dyslexia have difficulty learning to read and many lack fluent word recognition as adults. In a novel task that borrows elements of the ‘word superiority’ and ‘word inversion’ paradigms, we investigate whether holistic word recognition is impaired in dyslexia. In Experiment 1 students with dyslexia and controls judged the similarity of pairs of 6- and 7-letter words or pairs of words whose letters had been partially jumbled. The stimuli were presented in both upright and inverted form with orthographic regularity and orientation randomized from trial to trial. While both groups showed sensitivity to orthographic regularity, both word inversion and letter jumbling were more detrimental to skilled than dyslexic readers supporting the idea that the latter may read in a more analytic fashion. Experiment 2 employed the same task but using shorter, 4- and 5-letter words and a design where orthographic regularity and stimuli orientation was held constant within experimental blocks to encourage the use of either holistic or analytic processing. While there was no difference in reaction time between the dyslexic and control groups for inverted stimuli, the students with dyslexia were significantly slower than controls for upright stimuli. These findings suggest that holistic word recognition, which is largely based on the detection of orthographic regularity, is impaired in dyslexia.

## Introduction

Developmental dyslexia is characterised by marked difficulties in learning to read that cannot be attributed to low intelligence, poor motivation or to lack of education and opportunity to read [1]. In standardised measures of reading ability the scores of children with dyslexia are found to scatter on the lower tail of a normal distribution, suggesting that dyslexia is not a discrete or easily defined diagnostic entity [2]. Clinical reviews paint a similarly complex picture with noted overlap between dyslexia, language impairment and speech sound disorder [3].

Fluent word recognition—the effortless mapping between the sight of a printed word and its sound or pronunciation—is central to reading and develops through the acquisition of two distinct skills, phonological and orthographic coding [4]. Phonological coding refers to the representation of grapheme-phoneme correspondences–the matching between letters and their associated sounds—and is crucial to the reading of new and unfamiliar words. Orthographic coding refers to the representation of the visual form of words which includes letter combinations at both coarse and fine scales that signal spelling regularities and that help identify words without the need to access phonological information at the sub-word level [5]. Reading fluency reflects expertise in this visual or direct-access route to word sounds. For example, whereas the time to name a word increases with word length for children who are learning to read and still ‘sounding out’ the words, word naming latency is largely independent of word length in fluent adult readers [6].

Bruck [7]argues that poor phonological processing—a central characteristic of dyslexia [8]—prevents dyslexic readers from accurately noting spelling-sound correspondences in reading which, in turn, prevents them from learning precise orthographic information about words. Ironically, it is their poor knowledge of spelling-sound correspondences that keeps dyslexic readers using these very cues to recognize words, arresting their reading development and preventing them from eventually achieving fluency via the automatic use of orthographic or visual word form cues to recognize words and to access their sounds directly.

Bruck’s idea is an interesting one that long predates current knowledge of the neural basis of visual expertise in word recognition. The visual word form area (VWFA), located in mid-fusiform gyrus in the left hemisphere, is a region of the brain that is activated during reading and that responds preferentially to words over consonant strings, chequerboard patterns [9] and line drawings of objects [10]. Activation of the VWFA is constant across changes in the retinotopic location of word stimuli [9]and across changes in their typographic case and size [11,12]. This response invariance to stimulus properties that are unimportant to word recognition coupled with a robust response to pseudowords—non-words that mimic the spelling structure of real words—suggests that the VWFA represents words in a way that captures their orthographic regularity rather than their physical form or associated semantics [12,13]. In fact, in studies using fMRI adaptation the VWFA shows greater selectivity—to whole words rather than to sub-lexical orthographic patterns—suggesting that the region may serve as a form of ‘visual dictionary’ [14,15].

Decreased brain activity in the VWFA has been reported in dyslexia [16–18]. Using a cross-sectional design to investigate changes associated with reading development, Shaywitz and colleagues [17] report that while normal readers show increased activation in the VWFA with age, dyslexic readers instead show increased activation of a nearby (posterior medial occipitotemporal) region. They suggest that increased VWFA activation in normal readers reflects changes associated with increasing fluency in mapping word forms to associated sounds, whereas increased activation in the neighbouring region in dyslexic children reflects increasing reliance on memorization of the visual word form. Others [18] show that while the VWFA is specialized for orthographic regularity at both a coarse scale (distinguishing print from non-print fonts) and at a finer scale that taps into spelling regularity in normal readers, the VWFA in dyslexic readers does not make this distinction. Together these studies point to anomalous development of the VWFA in dyslexia and implicate impaired sensitivity to orthographic regularity as a source of impaired fluency in reading.

We suggest that this insensitivity to orthographic regularity in dyslexia might usefully be conceptualized as a problem in acquiring a particular type of perceptual expertise, one that relies on holistic processing of words. Broadly speaking, perceptual expertise involves discriminating instances of a category—such as recognizing individual words or faces—and is thought to engage distinct processes from those involved in more basic categorization as when we identify a visual object as a word or as a face [19]. As noted by a number of researchers, words and faces share a characteristic that poses a particular challenge for their individual identification, specifically, their high self-similarity [20,21]. Individual words are made by arranging a fixed number of letters from a limited alphabet and individual faces share a common arrangement of features with each other.

In the case of faces, identification of individuals is thought to involve holistic or configural processing [19] and a number of experimental paradigms provide support for this idea. In the ‘composite face effect’ careful alignment of the top half of one face with the bottom half of another leads us to perceive a novel facial configuration that slows or prevents recognition of the component faces [22]. Central to this effect is an inability to selectively attend to one part of a face and ignore information from other, a type of ‘obligatory attention’ to all parts of the stimulus [21,23]. The ‘face inversion effect’ links holistic processing to the upright orientation of a face and shows that face recognition [24] and sensitivity to distortions of facial shape [25] are compromised when faces are inverted. Finally, Tanaka and Farah’s variant of the ‘face superiority effect’—which shows that facial features are more easily recognized within the context of a face than when presented alone or within a face with spatially scrambled features [26]–also points to the importance of facial configuration in face recognition.

These hallmarks of expertise in face recognition–an obligatory attention to the configuration of facial features that is specific to the typical, upright orientation of faces, and a superior ability to recognize facial features within the context of a face—have parallels in the study of word recognition. The ‘obligatory attention’ to all parts of the stimulus that is central to the composite face effect has recently been demonstrated for words. Wong and colleagues [21] asked participants to match the right or left halves of a pair of words while ignoring the other halves on both congruent trials, (both halves of the pair of words are matched), or incongruent trials (one half of the pair is matched, the other is mismatched). They found that performance was better on congruent than incongruent trials, showing interference from the supposedly ignored halves of the words. This ‘composite word effect’ was stronger for highly familiar words, i.e., those from participants’ first compared to their second language and for real words compared to pseudo-words [21].

Secondly, there is evidence that words are read in a holistic manner over the range of orientations at which we normally encounter them. A seminal study of reading rotated words suggests that words are read ‘holistically’ at orientations at or close to the upright but in a more piecemeal or analytic fashion when rotated by 180 degrees [27]. Response times to recognize words are independent of word length between 0° and ~ 60°, increase with increased word length in a manner suggesting piecemeal reading of sub-lexical units, with a final shift to very slow letter by letter reading between beyond ~120°. This ‘word inversion effect’ holds for words presented in their familiar format but not for words written backwards or for mirror reversed words [28]. Finally, the face superiority effect has obvious parallels with the ‘word superiority effect’ which shows that the recognition of single letters is more efficient when they are embedded in real words than when presented alone or embedded in meaningless string of letters [29].

The current study utilizes design aspects of both the word inversion and the word superiority paradigm to investigate the idea that adult dyslexics are somehow less tuned to the visual form of words than skilled adult readers using a task that does not require reading. We asked participants to judge whether a pair of real words or a pair of letter strings (formed by jumbling the letters of the words) were the same or differed by a single letter. The word-superiority effect [29] predicts that performance should be best in the real word condition. Additionally, we asked participants to make this perceptual judgement for both upright words and letter strings and for the same stimuli which had been inverted. The ‘word inversion effect’ predicts for our study superior performance in the upright condition. To preview our results, we found that both adults with dyslexia and skilled adult readers preformed as predicted, showing fastest response times for upright real words and slowest for inverted letter strings. Most interestingly, the effects of inversion and degradation of orthographic regularity were more detrimental to the skilled readers than to those with dyslexia, supporting the idea that dyslexic readers may continue to read in a more analytic fashion into adulthood and never attain full automaticity through the exclusive use of visual word form cues [7].

## Experiment 1

### Methods

#### Participants

The experimental group comprised 30 students (17 females) with a mean age of 23.6 years (SD = 6.07 years) and age range of 18 to 43 years who were registered with University College Dublin (UCD) Disability Support Services as dyslexic, having provided evidence of diagnosis by an educational or clinical psychologist on registration. Diagnoses of dyslexia accepted by the university are based on analysis of performance on a battery of standardized norm-based tests including (a) word reading: fluency, speed and accuracy (b) word comprehension and spelling, (c) phonological awareness, (d) working memory and (e) rapid automatic naming. To qualify for special arrangements that are put in place by Disability Support Services students must score at or below the 10^th^ percentile on at least two literacy tests from the following (reading accuracy, reading speed, single word reading, spelling, reading comprehension, writing speed and pseudo-word decoding). Of the 30 participants recruited, 4 reported other diagnoses, 2 for Specific Language Impairment in childhood, and 1 for ADHD and 1 for Tourette Syndrome, both concurrent. The control group comprised 30 students (15 females) with a mean age of 23.1 years (SD = 5.07 years) and age range of 18 to 44 years, also from UCD. The groups did not differ significantly in age, t = 0.35, df = 56.23, *p* = 0.73. The study was approved by the Human Research Ethics Committee (HREC) at UCD (http://www.ucd.ie/researchethics); in accordance with the Declaration of Helsinki all participants gave written, informed consent and were advised of their right to withdraw from the study at any time without prejudice.

#### Stimuli

The word stimuli were generated from 15 high-frequency 6-letter and 15 high-frequency 7-letter single morpheme American English words taken from http://slpath.com/ and based on the Kučera & Francis database [30]. The 30 words are shown in Table 1 alongside their SUBTLEX frequency per million words [31] where they all, bar one, also qualify as ‘high-frequency’ using the criterion of a frequency higher than 10 per million. The stimuli were presented in pairs across 4 conditions (upright-real, upright-jumbled, inverted-real, inverted-jumbled) in a task in which participants judged whether the stimuli were the same or different.

**Table 1:** The 30 high frequency words used in Experiment 1 to generate the experimental stimuli alongside their SUBTLEX frequency per million words.

Six letter words	SUBTLEX frequency per million words	Seven letter words	SUBTLEX frequency per million words
**action**	61.08	**against**	201.73
**almost**	193.27	**already**	318.55
**Always**	655.25	**another**	509.22
**coarse**	1.35	**certain**	85.37
**either**	182.51	**country**	161.84
**enough**	501.33	**general**	115.39
**family**	354.25	**history**	83.92
**number**	240.94	**perhaps**	136.06
**public**	71.08	**present**	89.45
**rather**	114.22	**problem**	330.06
**result**	19.76	**quality**	18.57
**second**	284.57	**several**	38.78
**social**	33.39	**service**	79.92
**should**	1061.94	**through**	549.53
**system**	91.51	**weather**	34.24

In the upright-real condition, each word was either paired with itself onscreen (e.g., ‘another’, ‘another’) on the ‘same’ trials, or with a variant of itself in which a single letter was changed (e.g., ‘another’, ‘arother’) on the ‘different’ trials, the original word always appearing to the left and the modified or misspelled word to the right. Following [32], changes were made that best preserved the overall word shape using letter substitutions from their Table 2, with a further stipulation that substitutions were always made within the word, the first and last letter never changing. For the upright-jumbled condition, the same 30 words were used to create jumbled or scrambled letter strings, with the same letter substitutions being used on ‘different’ trials as were used in the upright-real condition, thus (‘another’, ‘another’) became (‘hanrtoe’, ‘hanrtoe’) for the equivalent ‘same’ trial and (‘hanrtoe’, ‘hahrtoe’) for the equivalent ‘different’ trial. Finally, in the inverted-real and inverted-jumbled conditions, the entire stimulus was rotated through 180° so that the letters were upside-down and the words reversed left to right.

**Table 2:** The 30 high frequency words used in Experiment 2 to generate the experimental stimuli alongside their SUBTLEX frequency per million words.

Four letter words	SUBTLEX frequency per million words	Five letter words	SUBTLEX frequency per million words
**area**	74.92	**argue**	19.75
**clue**	17.61	**baron**	13.47
**date**	141.53	**chase**	32.80
**drop**	130.61	**clown**	15.82
**dump**	28.82	**cloud**	11.75
**duck**	24.76	**enemy**	48.51
**dish**	11.45	**empty**	47.24
**exam**	13.41	**fault**	104.12
**foul**	14.47	**honor**	96.31
**fund**	10.61	**nurse**	44.98
**gold**	78.94	**prize**	22.39
**gate**	32.04	**pound**	13.88
**lawn**	12.35	**roses**	14.18
**rent**	34.55	**stage**	45.57
**soul**	76.96	**shell**	13.22

Each word pair was typed in Times New Roman lower case font, 80 pts, black on a white background, using Microsoft Powerpoint^®^, in two text boxes symmetrically spaced to either side of the slide centre. These were saved as JPEG images and the words subtended between ~6 and ~10 degrees of visual angle when presented onscreen at a comfortable viewing distance of ~60cm. The stimuli were presented and response time (RT) recorded using Presentation^®^ on a Dell PC with the display running at 60Hz and a spatial resolution of 1024 by 768 pixels.

#### Procedure

Each participant completed 240 trials, comprising the 30 distinct words by 4 conditions (upright-real, upright-jumbled, inverted-real, inverted-jumbled) by two trial types (same, different) and pressed either the M or Z key on the keyboard to indicate whether the stimulus pairs were the same or different. All 4 conditions were mixed within the block of 240 trials. After a short practice run of 16 trials in which participants were acquainted with all 4 conditions and prompted as to the correct response, they completed a further 24 practice trials without feedback. The 240 experimental trials were then presented in pseudo-random order with a short break after the first 120 trials. Participants were asked to respond as quickly as possible on each trial once they were reasonably sure of the correct answer, i.e., both speed and accuracy were stressed. They were assured that the task did not involve reading aloud, something that can be stressful for those with dyslexia.

### Results

The response time (RT) data were analysed in R [33] using ANOVA with a between-subjects factor of *Group* (dyslexic, control) and within-subjects factors of *Orientation* (upright, inverted), *Orthography* (real, jumbled) and *Word Length* (6-letter, 7-letter). A further measure of performance, sensitivity (*d’*), was analysed with factors of *Group* and *Condition*. Greenhouse-Geisser corrections were used when Mauchly's Test for Sphericity was significant and effect sizes are given by generalized eta squared (*η*^*2*^_*G*_) [34].

#### Response time

Response time (RT) analyses were carried out for correct trials only; 89.5% and 91.4% of trials for the experimental and control groups respectively. Exploratory data analyses highlighted a small number of outliers and 14 RTs (< 0.1% of all trials) greater than 16000ms or less than 600ms were removed.

**Fig 1:**
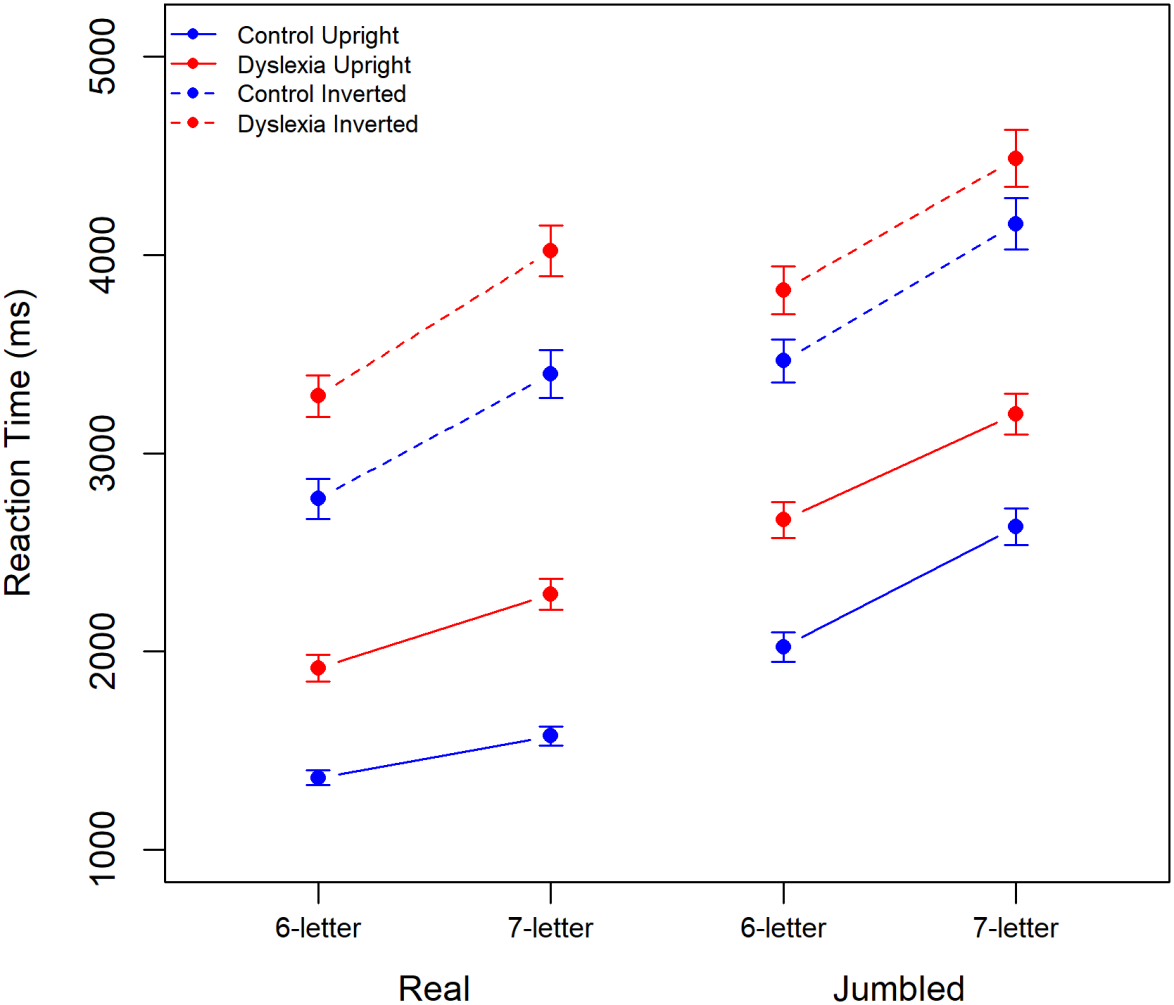
Mean RT for dyslexic and control participants for real (left) and jumbled (right) words with performance for 6- and 7-letter words and for upright and inverted stimuli plotted separately. Error bars show 95% CIs.

Mean response time (RT) is plotted for both dyslexic and control participants in all conditions in Fig 1. For both groups RT increases with word length and with increased task difficulty, being faster for upright than for inverted stimuli and for real words than for jumbled words. Control participants are notably faster than those with dyslexia across these conditions.

**Fig 2:**
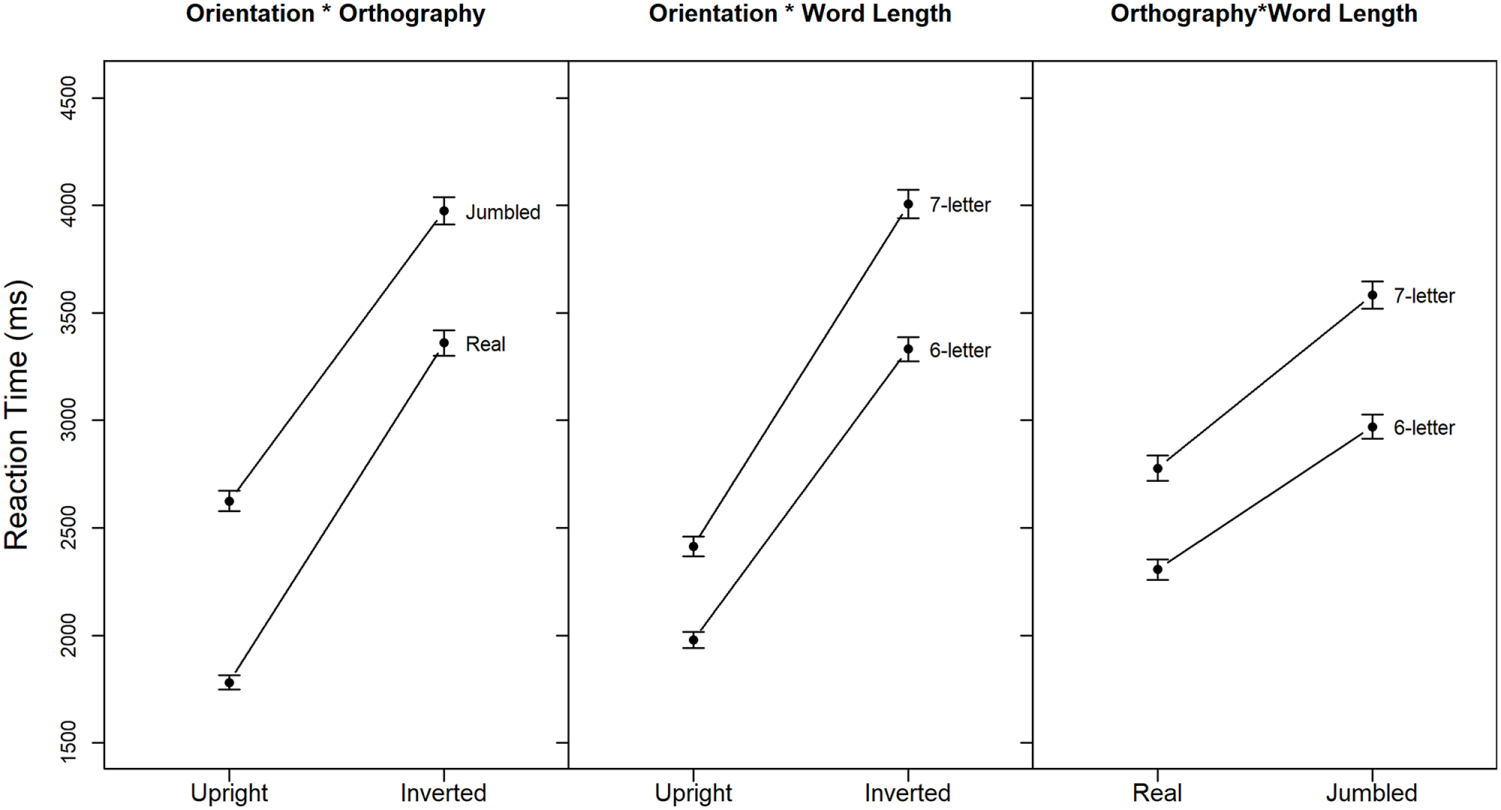
Mean response time (average across dyslexic and control participants) plotted to examine the three two-way interactions. Error bars show 95% CIs.

ANOVA with dependent variable of RT showed significant main effects of *Group*, F(1,58) = 7.51, *p* < 0.01, *η2G* = 0.09, of *Orientation*, F(1,58) = 329.81, *p* < 0.01, *η2G* = 0.41, of *Orthography*, F(1,58) = 272.61, *p* < 0.01, *η2G* = 0.15, and of *Word Length*, F(1,58) = 322.05, *p* < 0.01, *η2G* = 0.09. The analysis also revealed three significant two-way interactions: Orientation*Orthography, F(1,58) = 13.54, *p* < 0.01, *η2G* = 0.005, Orientation*Word Length, F(1,58) = 30.79, *p* < 0.01, *η2G* = 0.004, and Orthography*Word Length, F(1,58) = 11.08, *p* < 0.01, *η2G* = 0.002. These are easily interpreted from Fig 2, where the increase in RT with stimulus inversion is somewhat steeper for real than jumbled words and for 7-letter than 6-letter words and where jumbling of the letters within a word leads to steeper increase in RT for 7-letter than 6-letter words. As the effect of word length did not differ between the dyslexic and control participants, it was not explored in further analyses.

**Fig 3:**
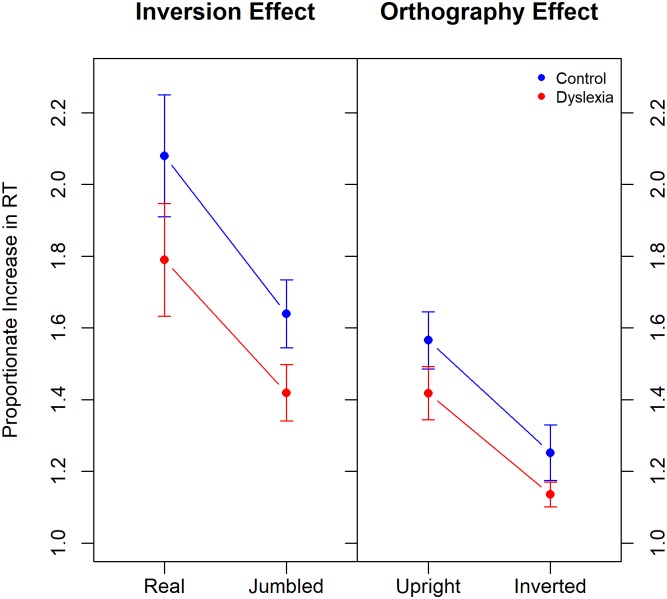
The mean proportionate increase in RT is plotted for dyslexic and control participants to demonstrate the effects of word inversion (left) and jumbling (right). Error bars show the 95% CIs.

To further explore the effects of ‘inversion’ and ‘orthography’, we calculated the proportionate increase in RT due to these experimental manipulations as follows. For the ‘inversion effect’ we calculated for each participant their mean RT in the inverted-real and inverted-jumbled conditions and divided by their corresponding mean RT for the upright-real and upright-jumbled conditions. For the ‘orthography’ effect we calculated for each participant their mean RT in the upright-jumbled and inverted-jumbled conditions and divided by their corresponding mean RT for the upright-real and inverted-real conditions. The mean proportionate increase in RT, for dyslexic and control participants, due to stimulus inversion and due to change in orthography is plotted in Fig 3. By these measures participants with dyslexia are, on average, less affected by stimulus inversion than are control participants and, similarly, they are less affected by changes in ‘orthography’—the jumbling of letters in the word- than are control participants.

Also evident from Fig 3 is that inversion is more detrimental to the processing of real than jumbled words and, similarly, that jumbling the letter order is more detrimental to the processing of upright than inverted words.

For the ‘inversion effect’ data ANOVA with between-subjects factor of *Group* and within-subjects factor of *Orthography* and dependent variable of proportionate increase in RT showed significant main effects of *Group* ($\overset{-}{X}$ = 1.60 [1.51, 1.70] for dyslexics, $\overset{-}{X}$ = 1.86 [1.75, 1.97] for controls), F(1,58) = 10.74, *p* < 0.01, *η2G* = 0.12, and of *Orthography* ($\overset{-}{X}$ = 1.53 [1.46, 1.60] for jumbled words, $\overset{-}{X}$ = 1.93 [1.82, 2.05] for real words), F(1,58) = 76.42, *p* ~ 0, *η2G* = 0.26. The Group* Orthography interaction was not significant (*p* = 0.45).

For the ‘orthography effect’ data ANOVA with between-subjects factor of *Group* and within-subjects factor of *Orientation* and dependent variable of proportionate increase in RT showed significant main effects of *Group*, ($\overset{-}{X}$ = 1.28 [1.22, 1.33] for dyslexics, $\overset{-}{X}$ = 1.41 [1.34, 1.48] for controls), F(1,58) = 15.80, *p* < 0.001, *η2G* = 0.12, and of *Orientation*, [$\overset{-}{X}$ = 1.19 [1.15, 1.24] for inverted words, $\overset{-}{X}$ = 1.49 [1.44, 1.55] for upright words), F(1,58) = 75.57, *p* ~ 0, *η2G* = 0.40. The Group*Orientation interaction was not significant (*p* = 0.65).

#### Sensitivity

**Fig 4:**
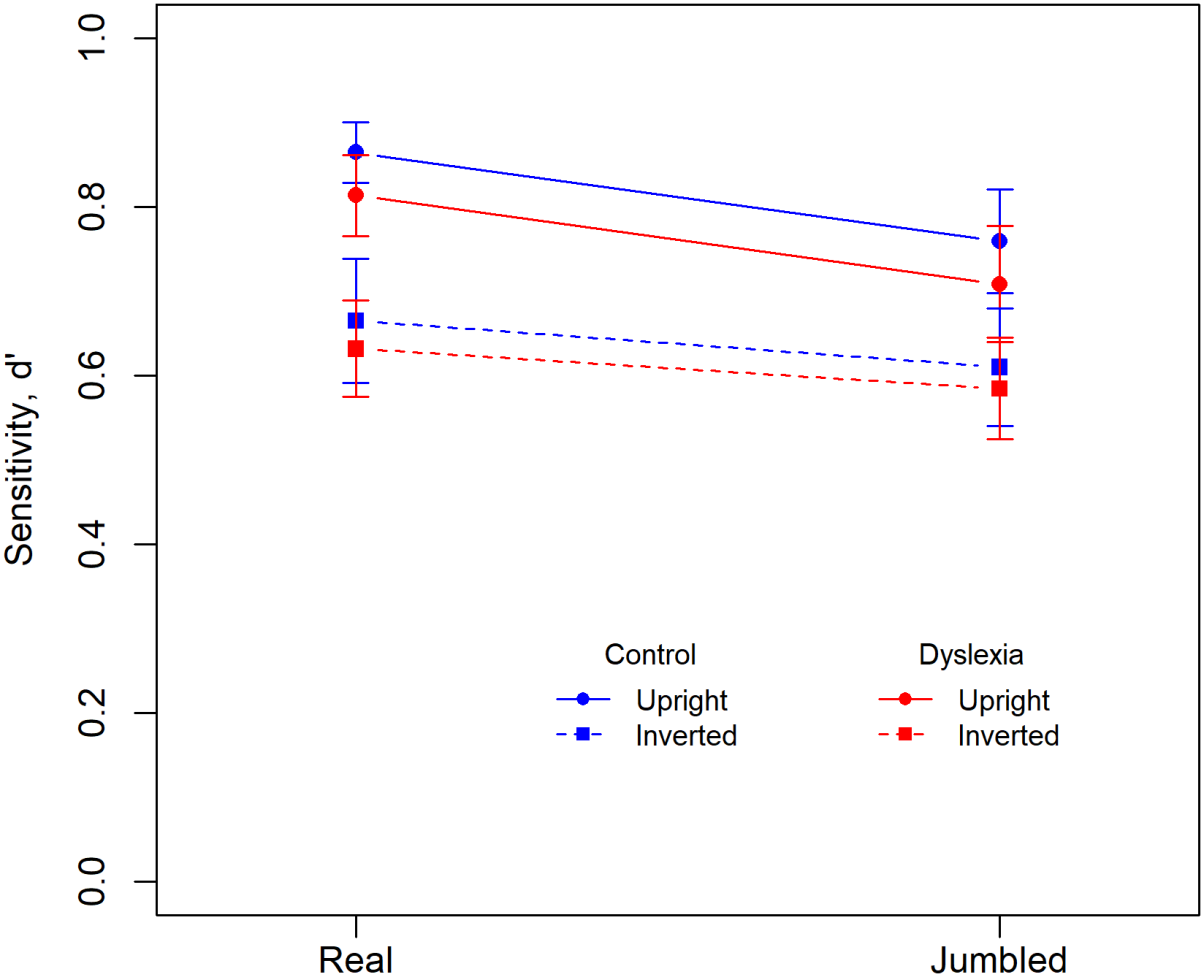
Mean sensitivity, d’, which has been scaled to a maximum value of 1.0, is plotted for all four stimulus conditions for dyslexic and control participants. Error bars show the 95% CIs.

Fig 4 plots *d’*—a measure of sensitivity that is independent of response bias calculated from the rates of ‘hits’ and ‘false alarms’ [35]–at each of the four stimulus presentation conditions with separate traces for the dyslexic and control participants, and where the *d’* data have been scaled to a maximum value of 1.0. Sensitivity declines gradually with increased task difficulty and while slightly lower for the dyslexic than control participants there is considerable overlap of the 95% CIs error bars for the two groups. ANOVA showed a significant main effect of *Condition*, F(3,174) = 59.52, *p* ~ 0, *η2G* = 0.25. Neither the main effect of *Group* (*p* = 0.25) nor the Group*Condition interaction (*p* = 0.88) were significant.

Planned post-hoc comparisons showed significant differences between the upright-real and upright-jumbled conditions, F(1,59) = 28.98, *p* ~ 0.0, *η2G* = 0.11, and between the inverted-real and inverted-jumbled conditions, F(1,59) = 10.76, *p* = 0.002, *η2G* = 0.02, showing that the effect of jumbling was significant for both upright and inverted words. Similarly, there were significant differences between the upright-real and inverted-real conditions, F(1,59) = 99.36, *p* ~ 0, *η2G* = 0.30, and between the upright-jumbled and inverted-jumbled conditions, F(1,59) = 43.63, *p* ~ 0, *η2G* = 0.14, showing that the effect of inversion was significant for both real and jumbled words. Bonferroni corrected alpha is 0.0125 for four comparisons.

### Discussion

Two general observations can be made about the results. First, the experimental task clearly engages perceptual expertise in word recognition, as response time increases with both inversion and with letter jumbling, establishing a gradient of difficulty whereby participants are fastest for upright-real words, next fastest for upright-jumbled words, slower again for inverted-real words and slowest for inverted-jumbled words. Although word length only varied by one letter, response times showed word length by orientation and word length by orthography interaction effects so that the latency differences for 6- and 7-letter words was greater for inverted than upright and for jumbled than real words. Therefore, the experimental manipulations served, as expected, to shift processing from an expert, largely holistic style to an increasingly analytic style and likely induced letter by letter reading in the most difficult condition. If participants were relying exclusively on an analytic strategy then word length effects would not be expected to vary with changing orientation and orthographic regularity.

Secondly, we stress that even the easiest condition likely involves some degree of analytic processing. This is because participants must scan between a pair of words in deciding whether they are the same or different. And, as spelling regularities exist at both a coarse, whole word scale and at finer sub-lexical scales [5], a strategy that capitalises on expertise for orthographic regularity may also involve some analytic processing.

Regarding the performance of dyslexic and normal readers, four aspects of the results are notable. First, the performance of both groups is consistent with the use of holistic processing, showing clear evidence of both superiority and inversion effects, i.e., participants from both groups are faster to detect a difference between the stimulus pairs when these are real words rather than jumbled words and when they are upright than inverted. Secondly, and central to this study, we find that college students with dyslexia are less hindered than their peers by changes in orientation and orthography that make the task more difficult. Specifically, the proportionate change in response time that accompanies both word inversion and word jumbling is significantly greater for normal readers than their dyslexic peers. This result is consistent with the idea that ‘compensated dyslexics’–adults who have successfully learned how to read–may read in a manner that still relies more on analytic and less on holistic processes. As such they may be less affected by the experimental conditions that force a shift from holistic to analytic processing. The third and fourth observations from our data are related, namely that the dyslexic college students are significantly slower than their peers in all conditions but show comparable sensitivity in the task. This is consistent with a recent study comparing the cognitive profile of dyslexic college students with their peers [36]; here differences in various performance measures, apart from spelling, reflect response time rather than accuracy differences.

In Experiment 2 we modified the task to address two limitations of the first study. First, there is the issue of word length. While the 6- and 7-letter words we used might not be classified as particularly ‘long words’ by some accounts [37], using shorter words may increase the use of holistic processing in the easier conditions. A second limitation of Experiment 1 relates to the use of a non-blocked design, i.e., all four conditions were intermixed so that participants, insofar as they used different strategies in the different conditions, could not predict in advance the optimal strategy from trial to trial. Therefore, Experiment 2 utilized shorter words of 4 or 5 letters and a blocked design with the expectation of revealing more marked differences between the groups.

## Experiment 2

### Methods

The methods were identical to those of Experiment 1 except where noted below.

#### Participants

The experimental group comprised 16 students (12 females) with a mean age of 21.0 years (SD = 3.18 years) and age range of 18 to 31 years who were registered with UCD Disability Support Services as dyslexic. Of the 16 participants recruited, 3 reported other diagnoses, 2 for ADHD and 1 for Autism Spectrum Disorder. The control group comprised 16 students (6 females) with a mean age of 22.69 years (SD = 1.45 years) and age range of 20 to 25 years, also from UCD. The groups did not differ significantly in age, t = 1.93, df = 20.95, *p* = 0.07. The study was approved by the Human Research Ethics Committee (HREC) at UCD (http://www.ucd.ie/researchethics); in accordance with the Declaration of Helsinki all participants gave written, informed consent and were advised of their right to withdraw from the study at any time without prejudice.

#### Stimuli

The word stimuli were generated from 15 high-frequency 4-letter and 5-letter words taken from the SUBTLEX database which reports the frequencies of words taken from the subtitles of American movies and television series (31). Four and five letter words from the corpus were initially filtered to include only those having a frequency higher than 10 per million. They were further filtered to exclude articles, prepositions, adjectives, adverbs, proper nouns, conjunctions, acronyms, foreign language words, informal or slang words, plurals, verbs in past or future tense and onomatopoeic words. A random number generator was then used to pick 30 words which are shown in Table 2 alongside their frequency per million words where they all qualify as ‘high-frequency’ using the criterion of a frequency higher than 10 per million.

#### Procedure

The procedure was identical to that in Experiment 1 except that stimuli from the 4 conditions (upright-real, upright-jumbled, inverted-real, inverted-jumbled) were presented in separate blocks with the order of the blocks presented in pseudo-random order across participants.

### Results

#### Response time

Response time (RT) analyses were carried out for correct trials; 91.30% and 94.14% of trials for the experimental and control groups respectively. A small number of outliers (< 0.17% of all trials) greater than 16000ms or less than 600ms were removed.

**Fig 5:**
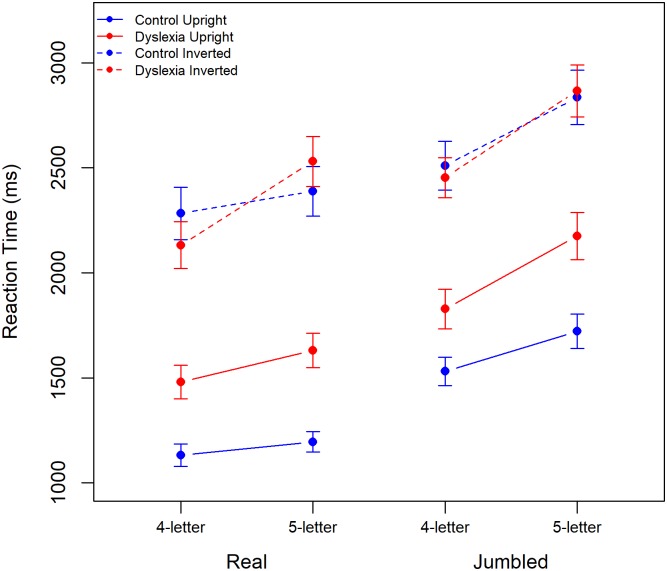
Mean RT for dyslexic and control participants for real (left) and jumbled (right) words with performance for 4- and 5-letter words and for upright and inverted stimuli plotted separately. Error bars show 95% CIs.

Mean response time (RT) is plotted for both dyslexic and control participants in all conditions in Fig 5. For both groups RT increases with word length and with increased task difficulty, being faster for upright than for inverted stimuli and for real words than for jumbled words. Control participants are notably faster than those with dyslexia in the upright but not in the inverted stimulus conditions.

ANOVA with dependent variable of RT showed significant main effects of *Orientation*, F(1,30) = 106.69, *p* < 0.001, *η2G* = 0.38, of *Orthography*, F(1,30) = 54.07, *p* < 0.001, *η2G* = 0.11, and of *Word Length*, F(1,30) = 92.51, *p* < 0.001, *η2G* = 0.04. The analysis also revealed four significant two-way interactions: Word Length*Orthography, F(1,30) = 7.51, *p* < 0.02, *η2G* = 0.003, Word Length*Orientation, F(1,30) = 20.24, *p* < 0.01, *η2G* = 0.003, Word Length*Group, F(1,30) = 8.66, *p* < 0.01, *η2G* = 0.004, and Group*Orientation, F(1,30) = 5.29, *p* <0.05, *η2G* = 0.03.

**Fig 6:**
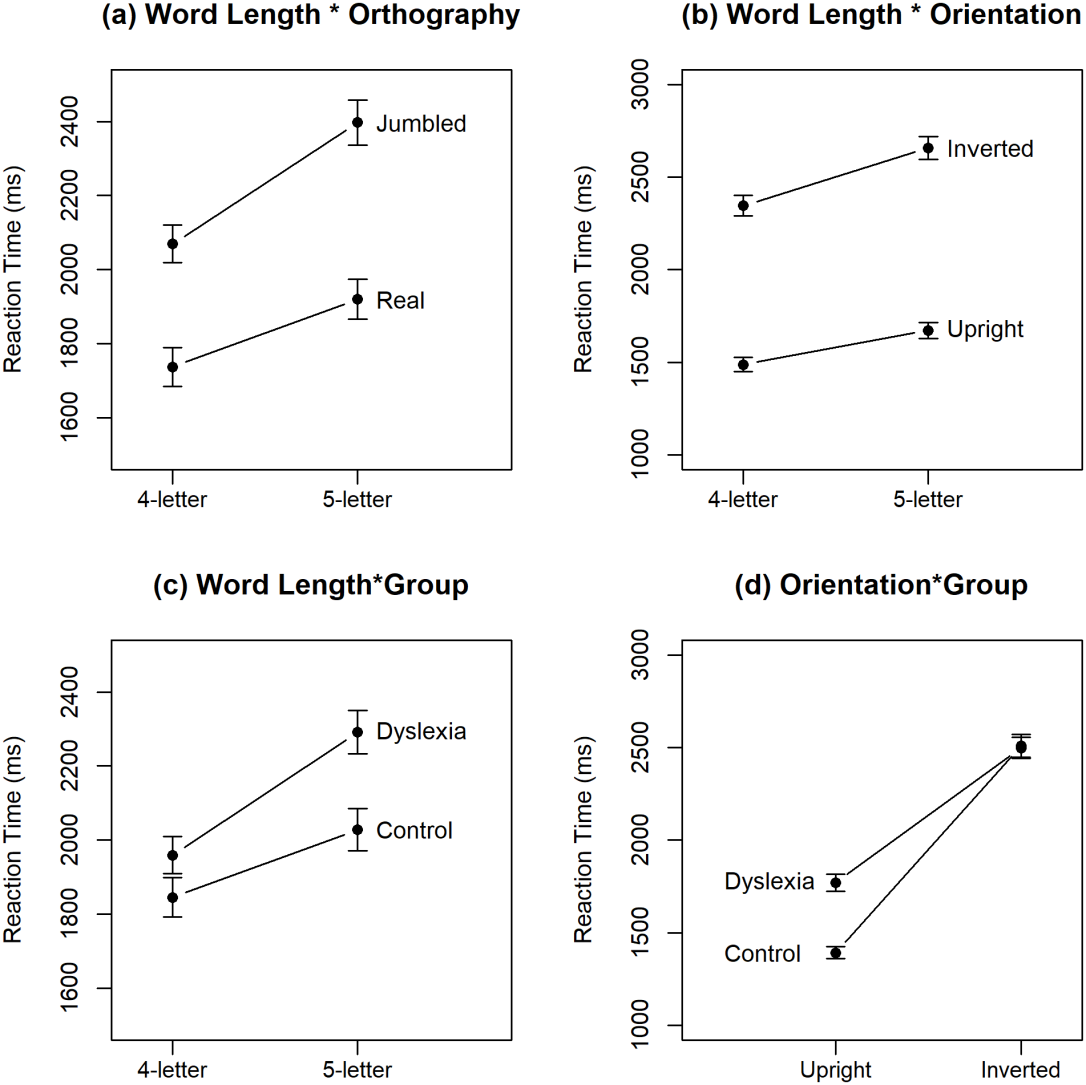
Mean response time plotted to examine the four two-way interactions. Error bars show 95% CIs.

These are shown in Fig 6 where (a) the increase in RT with word length is more marked for jumbled, $\overset{-}{X}$ = 306.75ms [228.02,385.48], than real words, $\overset{-}{X}$ = 180.85ms [111.58,250.13], and (b) for inverted, $\overset{-}{X}$ = 305.85ms [230.49,381.22], than for upright, $\overset{-}{X}$ = 181.75ms [130.59,232.91], words. Of most interest is the effect of group. While RT is significantly greater for 5-letter than for 4-letter words for both the dyslexic and control samples (c), the increase in RT is more marked for the dyslexic, $\overset{-}{X}$ = 318.41ms [242.44,394.37], than for the control, $\overset{-}{X}$ = 169.20ms [92.36,246.04], group. Finally, looking to the effect of stimulus orientation in panel (d), while this is significant for both groups, the increase in RT that accompanies stimulus inversion is more marked for the control, $\overset{-}{X}$ = 1103.13 [819.60,1386.66], than for the dyslexic, $\overset{-}{X}$ = 701.32 [459.96, 942.69], group. Alternatively, looking to the RT difference between the two groups, this is significant for upright stimuli, F(1,30) = 5.48, p = 0.026, with the 95% CIs on the difference estimate as [43.66, 731.93], but not for the inverted stimuli, F(1,30) = 0.004, p = 0.95, with the 95% CIs on the difference estimate as [-462.79, 434.78].

#### Sensitivity

**Fig 7:**
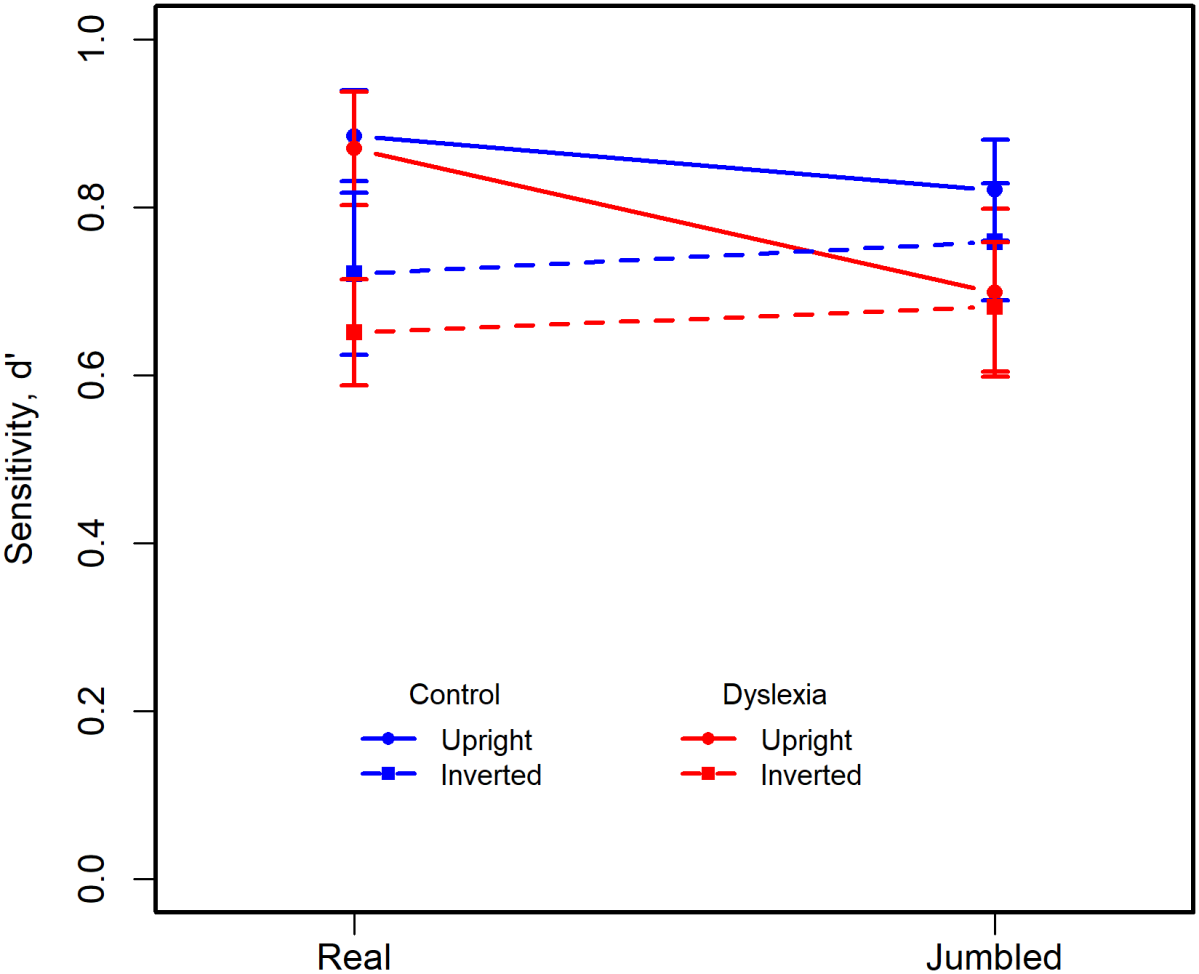
Mean sensitivity, d’, which has been scaled to a maximum of 1.0, is plotted for all four stimulus conditions for dyslexic and control participants. Error bars show the 95% CIs.

Fig 7 plots sensitivity *d’* at each of the four stimulus presentation conditions for both dyslexic and control participants. Sensitivity declines gradually with increased task difficulty and while slightly lower for the dyslexic than control participants there is considerable overlap of the 95% CIs error bars for the two groups. ANOVA showed a significant main effect of *Condition*, F(3,90) = 20.45, *p* ~ 0, *η2G* = 0.22. Neither the main effect of *Group* (*p* = 0.07) nor the Group*Condition interaction (*p* = 0.25) were significant.

Planned post-hoc comparisons showed significant differences between the upright-real and upright-jumbled conditions, F(1,31) = 14.66, *p* < 0.001, *η2G* = 0.15, but not between the inverted-real and inverted-jumbled conditions, F(1,31) = 2.14, *p* = 0.15, *η2G* = 0.01, showing that the effect of jumbling was significant only for upright words. Similarly, there was a significant difference between the upright-real and inverted-real conditions, F(1,31) = 51.49, *p* ~ 0, *η2G* = 0.34, but the difference between the upright-jumbled and inverted-jumbled conditions did not reach significance at conventional levels, F(1,31) = 3.09, *p* = 0.09, *η2G* = 0.02, showing that the effect of inversion was significant for real words only. Bonferroni corrected alpha is 0.0125 for four comparisons.

### Discussion

Using the same task as in Experiment 1 but with shorter words and a design which facilitates the use of a fixed perceptual strategy within experimental blocks, Experiment 2 extends and strengthens our findings. Specifically, whereas differences between dyslexic and control participants in Experiment 1 were only evident in the analysis of the proportionate increase in RT, the reaction time analysis from Experiment 2 shows two significant interactions involving experimental group.

First, the groups differ in how they are affected by word length; dyslexic participants show a more marked increase in RT for longer over shorter words suggesting a greater reliance on analytic processing. Secondly, while stimulus inversion–known to impair holistic processing of both faces [38]and words [28]—leads to an increase in RT for both groups this increase is more marked for control than dyslexic participants. Specifically, while the groups do not differ in RT for inverted stimuli, the control group are significantly faster than the dyslexic group in judging upright stimuli suggesting that they benefit particularly from holistic processing of words and word-like stimuli.

Experiment 2 also shows significant interactions of word length with stimulus orientation and of word length with orthographic regularity, such that word length effects are greater for inverted than upright stimuli and for jumbled than normal stimuli. As in Experiment 1, this suggests that the experimental manipulations worked as expected.

## General discussion

The word superiority effect attests to perceptual expertise in word recognition whereby familiar words are read in a largely holistic fashion [29,39]. This sensitivity to orthographic regularity—to the visual form of familiar words—is tied to the orientation of words that we encounter while reading [27,28] just as holistic processing of faces is tied to orientations at or near the upright [40]. This same perceptual expertise is engaged in our study in which participants judge whether pairs of words or pairs of letter strings are the same or different and where the stimuli are presented in both upright and inverted form.

In both Experiments 1 and 2, the experimental manipulations of stimulus inversion and degradation of orthographic regularity worked to induce a shift from a predominantly global or holistic style of processing to one that is more local or analytic. And in both experiments, RT differences between the groups suggest that those with dyslexia may rely more on a local analysis of words and word-like stimuli as their default processing style. As such they are less affected by the experimental manipulations that their non-dyslexic peers. In Experiment 1 these differences only emerge in an analysis of the proportionate increase in RT that accompany changes in orthographic regularity and stimulus orientation, with dyslexic participants being comparatively less affected as the task becomes more difficult. While consistent with the general thesis that dyslexics may rely more on analytic processing in processing words and word-like stimuli, this conclusion is strengthened by the results of Experiment 2 which shows significant interactions of experimental group with both word length and stimulus orientation, both indicative of a greater reliance on analytic processing in the dyslexic group.

The second experiment differed from the first in two respects, namely in the use of shorter words and a blocked design, both manipulations used to encourage the use of holistic processing. A comprehensive review and reanalysis of word length effects in word recognition which controls for word frequency effects [37] found that that word length is facilitatory for short words of 3–5 letters, does not affect performance for words of 5–8 letters and is inhibitory for longer words. While this suggests that the 6- and 7-letter words we used, especially as they are highly familiar words, might not be classified as particularly long words, shortening word length in Experiment 2 clearly had effect of highlighting differences between the groups.

Regarding the use of a blocked design in Experiment 2, recent research on discrimination of both simple tones and more complex speech sounds demonstrate that a key factor distinguishing dyslexic and control participants in such sound discrimination tasks is the ability, on the part of controls, to take advantage of a ‘perceptual anchor’ [41]. Specifically, whereas the two groups show no difference in sound discrimination under conditions in which there is no common reference stimulus on successive trials, the presence of such a perceptual anchor (e.g., on each trial participants are judging whether a test tone is higher or lower than a common reference tone of fixed frequency) leads to a strong advantage to non-dyslexic participants. Thus, the use of a blocked design may also have contributed to highlighting groups differences in Experiment 2.

In relating our findings to those of previous researchers, we recap the main findings. First, both groups show word superiority and word inversion effects, being faster to judge the similarity of upright than inverted stimuli and for real than for jumbled words. Previous research with dyslexic children (mean age, 11.5 years) reports robust word superiority and pseudo-word superiority effects in the face of compromised phonological skills [42]. As the children failed to show a word superiority effect of greater magnitude than the pseudo-word superiority effect, the authors argue that the effects reflect sensitivity to orthographic regularity rather than to lexical processes. Reilhac and colleagues [43] used a different task in which dyslexic children and controls had to decide whether two sequentially presented 4-letter strings (including words, pseudowords and non-words) were the same or different and where the differences included letter substitutions or letter transpositions. When the difference between pairs of letter strings involved letter substitution, the dyslexic children showed an advantage for both real words and pseudowords over non-words but they did not show a WSE above and beyond the PWSE, again arguing for intact sub-lexical processing of word forms in dyslexia. Similarly, our results with dyslexic college students point to intact sensitivity to the visual form of the word, with increased response times when the word form is degraded by jumbling or by inversion.

Secondly, college students with dyslexia are less hindered than their peers by changes in orientation and orthography and more hindered by increased word length, all factors that make the task more difficult. This is evident from Experiment 1 where the proportionate change in response time that accompanies both word inversion and word jumbling is significantly greater for normal readers than their dyslexic peers. And in Experiment 2 those with dyslexia are more affected by increases in word length but less affected by changes in stimulus orientation than the control group. These finding are consistent with the idea that adults with dyslexia may read in a manner that still relies more on analytic and less on holistic processes. In the case of reading, Bruck [7] has argued that college students with dyslexia show ‘arrested’ development and rely on strategies typical of much younger readers, specifically using more spelling-sound correspondences and context to recognize individual words. Although our task does not involve explicit reading or word recognition, one interpretation of the results is that they reflect a relatively greater reliance on analytic strategies in the dyslexic group and a poorer use of the cues of orthographic regularity that are of greater benefit to the normal readers.

While caution must be taken in speculating about the neural basis of these difference, it is interesting that there are a number of reports of anomalous functioning of the VWFA in dyslexia [17,18], a region of the brain that represents the visual form of the word at a level that captures orthographic regularity and that is sensitive to word inversion [44]. In particular, the research of [17] suggests that a regions of the brain neighbouring the VWFA which is involved in memorization of the visual word form compensate for this lack of sensitivity to orthographic regularity in dyslexia.

Turning to the broader behavioural literature, we note that there are reports of superior orthographic processing in dyslexia. Using a large sample, Siegel and colleagues [45] compared dyslexic and normal readers at eight different reading levels on both an ‘orthographic awareness task’ which measured readers knowledge of the probable sequences of letters within words, and a ‘phonological awareness’ task which involved reading pseudo-words aloud. Replicating many previous studies that show poorer phonological skills in dyslexia, this study also showed superior orthographic skills suggesting that dyslexic readers rely more on visual strategies and visual memory in learning to read. However, superior orthographic skills may not imply perceptual expertise in the sense of automatic or holistic recognition of word forms, e.g., while college students with dyslexia prefer a visual over a phonological strategy in matching words and non-words, this often includes a letter by letter visual scanning strategy [46]. And there is evidence that some aspects of orthographic knowledge, specifically letter position, is poorly developed in dyslexic children [43].

The final observations from our study is that college students with dyslexia are generally slower but show comparable sensitivity to their peers. As noted above, this is consistent with studies comparing the cognitive profile of dyslexic college students with their peers [36] where differences generally reflect response time rather than accuracy differences. Similarly, in the learning of non-words via practice and repetition, college students are reported to be slower but not less accurate than their peers [47] As a university education increases vocabulary and introduces subject-specific terminology that may present challenges for even the most accomplished dyslexic readers [1], knowledge of how ‘compensated’ dyslexics differ from their peers is crucial to providing supports at university. Persistent difficulties in reading are reported in college students with dyslexia, coupled with differences in a number of verbally based performance measures including working memory, arithmetic and tasks involving the retrieval of verbal information from long term memory [36,47] As such, the generally slower performance of the dyslexic participants in our study is perhaps not surprising.

Although generally slower, the dyslexic participants are less affected than their peers when experimental conditions force a shift from holistic processing to a style that favours analytic processing. One interpretation of these results is that while those with dyslexia are clearly sensitive to orthographic regularity, this expertise appears to be less well developed than in those with typical reading skills. This is consistent with what we know about dyslexia—that it is a disorder characterised by a lack of fluency, where ‘fluency’ reflects expertise in translating between the visual form of a word and its pronunciation without recourse to phonological information at the sub-lexical level [5] By some accounts reading fluency is synonymous with holistic visual processing, and it has been suggested that fluent readers can automatically recognize ~ 300 words that make up about 85% of those encountered in everyday reading [48].

More generally, while some have argued that word recognition involves predominantly part-based processing [49,50], a number of phenomena associated with reading and word recognition—including the word inversion effect [27,28,44], the word superiority effect [29,39], the word inferiority effect [51,52], our sensitivity to letter position in words [53], and the ‘illusory letter phenomenon’ [54]—point to a considerable role for holistic processing. And, as in face recognition, the use of ‘holistic’ information demonstrated by these phenomena is a marker of perceptual expertise in word recognition [21].

Relatedly, and in contrast to the idea that word recognition is a process whose efficiency is limited by having to recognize individual letters [49], metrics of capacity based on response times suggest that word recognition reflects a particularly efficient form of perceptual processing [55]. This efficiency may reflect in part the contribution of fast-acting, magnocellular processes that return an initial coarse analysis of word shape which facilitates perception at a configural or holistic level [56]. Interestingly, Chinese children with developmental dyslexia who show deficits in magnocellular visual processing fail to show the typical global over local processing advantage in the processing of Chinese characters that is shown by typical Chinese child readers. And this global advantage is intact in Chinese children with developmental dyslexia who show normal magnocellular visual processing.

To summarize, we introduce a novel task that does not require reading and that borrows design elements from both the word superiority and word inversion paradigms. We find that college students with dyslexia perform in a manner that suggests sensitivity to orthographic regularity showing increased response times when words are inverted or their letters jumbled. However, relative to their peers, they are less disadvantaged than their peers when forced to rely more on analytic processes in the task, suggesting comparatively poorer tuning to orthographic regularity. This is especially interesting in light of reports of hypoactivation in the VWFA in dyslexia [17,18], a region of the brain that gradually attunes itself to orthographic regularity as children learn to read [57]. While much research on dyslexia focuses on poor phonological processing, research on the visual skills of dyslexics, particularly their sensitivity to the redundancy inherent in visual word forms, offers new ways to study the disorder.
